# USC*γ* Dominated Community Composition and Cooccurrence Network of Methanotrophs and Bacteria in Subterranean Karst Caves

**DOI:** 10.1128/spectrum.00820-21

**Published:** 2021-08-18

**Authors:** Xiao-Yu Cheng, Xiao-Yan Liu, Hong-Mei Wang, Chun-Tian Su, Rui Zhao, Paul L. E. Bodelier, Wei-Qi Wang, Li-Yuan Ma, Xiao-Lu Lu

**Affiliations:** a State Key Laboratory of Biogeology and Environmental Geology, China University of Geosciences, Wuhan, China; b School of Environmental Studies, China University of Geosciences, Wuhan, China; c Institute of Karst Geology, CAGS/Key Laboratory of Karst Dynamics, MNR & GZAR, Guilin, China; d School of Marine Science and Policy, University of Delaware, Lewes, Delaware, USA; e Department of Microbial Ecology, Netherlands Institute of Ecology (NIOO-KNAW), Wageningen, the Netherlands; University of Minnesota

**Keywords:** Karst cave, atmospheric methane-oxidizing bacteria, cooccurrence network, subsurface biosphere, methane sink

## Abstract

Karst caves have recently been demonstrated to act as a sink for atmospheric methane, due in part to consumption by microbes residing in caves that can oxidize methane at atmospheric levels. However, our knowledge about the responsible atmospheric methane-oxidizing bacteria (atmMOB) in this vast habitat remains limited to date. To address this issue, weathered rock samples from three karst caves were collected in Guilin City and subjected to high-throughput sequencing of *pmoA* and 16S rRNA genes. The results showed that members of the high-affinity upland soil cluster (USC), especially upland soil cluster gamma (USC*γ*), with absolute abundances of 10^4^ to 10^9^ copies · g^−1^ dry sample, dominated the atmMOB communities, while *Proteobacteria* and *Actinobacteria* dominated the overall bacterial communities. Moreover, USC*γ* was a keystone taxon in cooccurrence networks of both the atmMOB and the total bacterial community, whereas keystone taxa in the bacterial network also included *Gaiella* and *Aciditerrimonas*. Positive links overwhelmingly dominated the cooccurrence networks of both atmMOB and the total bacterial community, indicating a consistent response to environmental disturbances. Our study shed new insights on the diversity and abundances underlining atmMOB and total bacterial communities and on microbial interactions in subterranean karst caves, which increased our understanding about USC and supported karst caves as a methane sink.

**IMPORTANCE** Karst caves have recently been demonstrated to be a potential atmospheric methane sink, presumably due to consumption by methane-oxidizing bacteria. However, the sparse knowledge about the diversity, distribution, and community interactions of methanotrophs requires us to seek further understanding of the ecological significance of methane oxidation in these ecosystems. Our *pmoA* high-throughput results from weathered rock samples from three karst caves in Guilin City confirm the wide occurrence of atmospheric methane-oxidizing bacteria in this habitat, especially those affiliated with the upland soil cluster, with a gene copy number of 10^4^ to 10^9^ copies per gram dry sample. Methanotrophs and the total bacterial communities had more positive than negative interactions with each other as indicated by the cooccurrence network, suggesting their consistent response to environmental disturbance. Our results solidly support caves as an atmospheric methane sink, and they contribute to a comprehensive understanding of the diversity, distribution, and interactions of microbial communities in subsurface karst caves.

## INTRODUCTION

Karst caves are characterized by permanent darkness, stable temperature, high humidity, oligotrophic conditions, and geographical isolation (1, 2) and are considered extreme environments. Recently, they have been demonstrated to be potential sinks for atmospheric methane (CH_4_), similar to upland soils (3, 4), mainly due to the widespread phenomenon that CH_4_ concentrations in caves are consistently below the contemporary atmospheric level (1.8 to 2.0 ppm) (3, 5, 6). Moreover, these subatmospheric CH_4_ concentrations in caves are attributed to the consumption of methane-oxidizing bacteria (MOB), as indicated by stable isotope analysis of methane (4, 7, 8). Known atmospheric methane-oxidizing bacteria (atmMOB) have the capacity of oxidizing subatmospheric levels of CH_4_ due to their high affinities for CH_4_ and are phylogenetically affiliated with upland soil cluster gamma (USC*γ*) and alpha (USC*α*) (9), which are widely distributed in various upland soil environments (9 to 11). Members of the upland soil cluster (USC) have been confirmed to be actively involved in CH_4_ oxidation under atmospheric and low CH_4_ concentrations (2 ppm and 20 ppm) (12) and have been demonstrated to be responsible for the oxidation of atmospheric CH_4_ (13–15).

Members of the atmMOB are resistant to cultivation, which gives rise to great difficulty in studying their physiology. To date, USC*γ* has no cultured representatives, and a single draft genome from it has been reported, which showed a close relationship with *Methylocaldum* (16). *Methylocapsa gorgona* MG08, affiliated with USC*α* Jasper Ridge 1 in a phylogenetic analysis of PmoA (amino acid sequence of the *pmoA* gene) and closely clustered with “*Candidatus* Methyloaffinis” via the analysis of the 16S rRNA gene, was isolated from the cover soil of a retired subarctic landfill (17). Fortunately, the functional gene encoding the beta subunit of particulate methane monooxygenase (pMMO), *pmoA*, is conserved in most MOB, except that *Methylocella*, *Methyloferula*, and *Methyloceanibacter* solely use the soluble methane monooxygenase (sMMO) (18–20). Therefore, *pmoA* can serve as an excellent phylogenetic marker to study the diversity of MOB (10, 21), and specific primers targeting *pmoA* of atmMOB have also been designed to detect their occurrence in natural ecosystems (21). Sequences of this gene have also been retrieved from the Heshang Cave in central China, showing that USC*γ* dominated the MOB communities in the weathered rocks (22). Therefore, sequencing and quantification of the *pmoA* gene exclusive to atmMOB are reliable approaches to characterize the overall diversity and abundance of atmMOB in subsurface karst caves.

The community structure of atmMOB in soil was demonstrated to be significantly controlled by pH: USC*α* generally dominates under acidic conditions, while USC*γ* prefers to live in neutral and alkaline habitats (9, 10, 23). Despite the role of pH in the distribution of atmMOB, pH does not affect the methane oxidation rate directly but, rather, acts on the abundance of MOB (24). Due to the alkaline conditions in karst caves (25), we assume that USC*γ* would dominate atmMOB communities. The CH_4_ concentration can also affect the capacity for atmospheric CH_4_ oxidation by atmMOB. Exposure to an increased CH_4_ concentration (∼10 ppm) increased the atmospheric CH_4_ oxidation rates in forest soils, where the MOB communities are mainly dominated by USC*γ* and USC*α* (26). Concentration gradients of CH_4_, as the direct substrate of microbial CH_4_ oxidation, are known to exist in numerous caves (3, 4). However, how the CH_4_ concentration impacts atmMOB communities in subsurface caves has remained mysterious.

**FIG 1:**
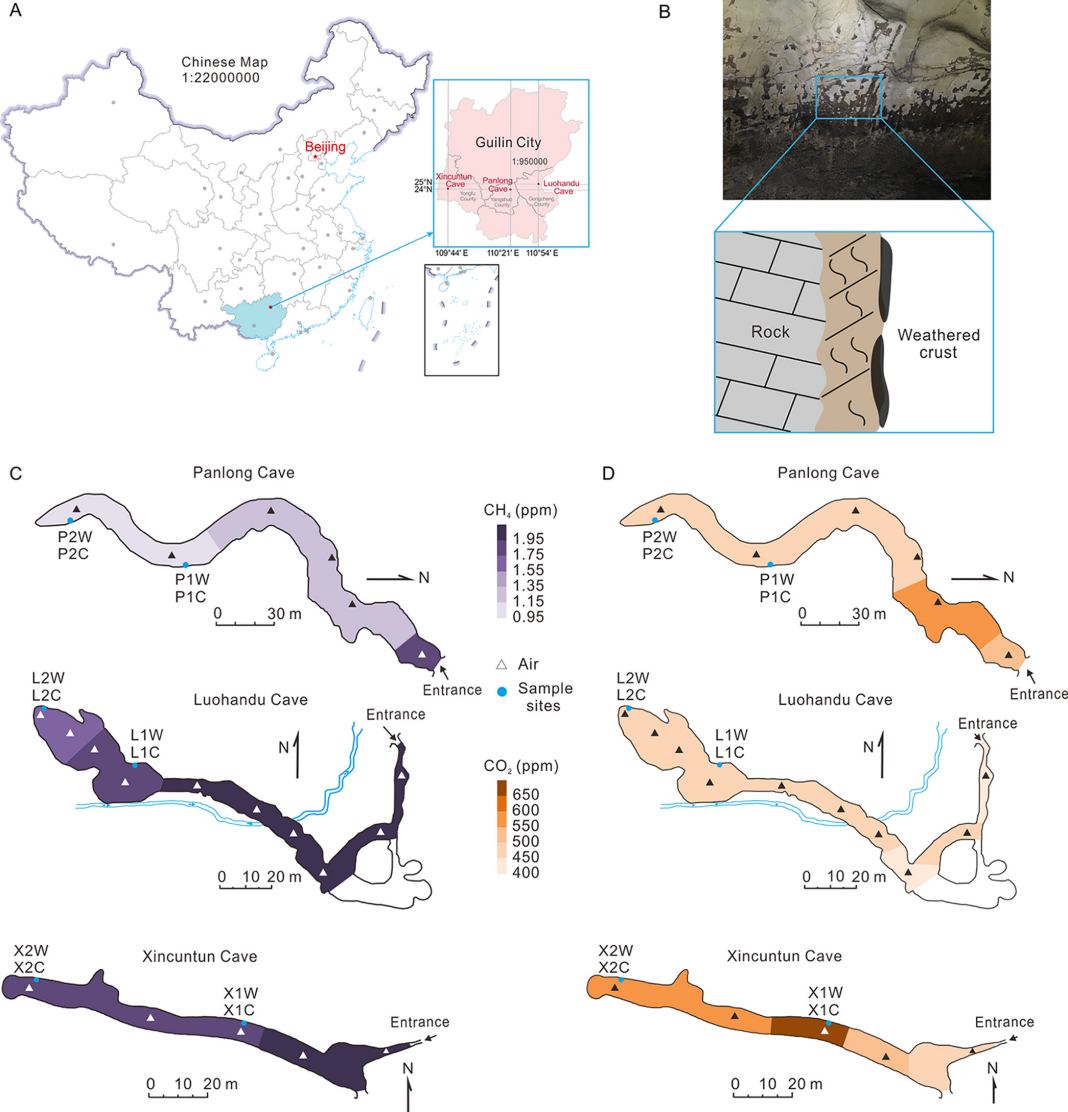
Geographic locations and atmospheric gas concentrations of the three karst caves investigated in Guilin City, southwest China. (A) The geographic locations of Panlong Cave, Luohandu Cave, and Xincuntun Cave on the map of China and Guilin City. The Chinese map was modified after http://bzdt.ch.mnr.gov.cn/. (B) Schematic diagram of weathered rocks. Spatial variability of the concentrations (ppm) of CH_4_ (C) and CO_2_ (D) in the cave atmosphere. Sampling sites within the caves are shown by blue dots, and air sampling sites are marked with triangles. P, Panlong Cave; X, Xincuntun Cave; L, Luohandu Cave; 1, sampling site at the middle of the cave; 2, sampling site far from the entrance of the cave; W, weathered rock; C, weathered crust.

To address these issues, we collected weathered rock samples and weathered crust samples from three different caves across Guilin City, Guangxi Province, southwestern China (Fig. 1). The samples were subsequently sequenced for *pmoA* and 16S rRNA genes via high-throughput sequencing. The aims of this study were to (i) investigate the abundance and distribution of atmMOB and other bacteria, (ii) explore the correlations between environmental factors and the total bacterial and atmMOB communities, and (iii) understand the cooccurrence patterns among bacterial taxa and MOB clades in weathered rock samples from subterranean caves. Our results will expand our understanding of the diversity and distribution of atmMOB in subsurface karst caves and the interactions between MOB/bacteria and environmental variables.

## RESULTS

### Physicochemical properties of weathered rock and crust samples.

Samples collected from the three karst caves in Guilin City (Panlong Cave [PLD], Luohandu Cave [LHD], and Xincuntun Cave [XCT]) (Fig. 1) were slightly alkaline or alkaline, with pH varying in the range of 7.78 to 9.56 (Table 1). The physicochemical properties varied with sample type (i.e., weathered rock versus crust), as well as the distance to the cave entrance. Specifically, the SO_4_^2−^ concentration varied with the distance to the cave entrance (Table 1). The pH and the concentrations of Cl^−^, K^+^, and Na^+^ varied with sample types in PLD and XCT, whereas these physicochemical parameters varied with sampling locations in LHD (Table 1). The weathering indices, such as the Ca/Si and Mg/Si ratios, of PLD were significantly different with the distance to the cave entrance. In contrast, the weathering indices in LHD and XCT were linked to niches (Table 1).

**TABLE 1:** Physicochemical properties and high-affinity USC gene copy numbers of weathered rock samples in three karst caves, Guilin City, southwestern China

Sample^*a*^	Mean abundance ± SD (copies · g^−1^ dry wt) (*n* = 108, contains 9 replicates) of USC^*b*^:		Mean value ± SD (*n* = 36, contains 3 replicates)^*c*^						
	USC*α*	USC*γ*	pH	Cl^−^ (mg·kg^−1^)	SO_4_^2−^ (mg·kg^−1^)	K^+^ (mg·kg^−1^)	Na^+^ (mg·kg^−1^)	Ca/Si ratio	Mg/Si ratio
P1W	3.01 × 10^6^ ± 1.26	3.35 × 10^8^ ± 1.24	9.19 ± 0.03a	2.36 ± 0.43a	21.72 ± 0.26a	5.04 ± 5.06a	6.95 ± 1.25a	3.09 ± 0.23a	0.88 ± 0.07a
P1C	1.49 × 10^6^ ± 1.18	5.68 × 10^8^ ± 1.46	7.78 ± 0.01b	39.04 ± 0.40b	31.82 ± 4.67b	20.90 ± 0.47a	12.63 ± 0.45a	3.04 ± 0.74b	0.12 ± 0.03b
P2W	4.58 × 10^4^ ± 1.63	6.78 × 10^5^ ± 1.56	9.26 ± 0.04a	4.37 ± 0.03c	57.37 ± 0.16c	0.71 ± 0.25a	7.77 ± 1.40a	37.25 ± 1.26a	5.76 ± 0.21a
P2C	1.44 × 10^8^ ± 1.24	4.37 × 10^8^ ± 1.13	8.07 ± 0.01c	26.70 ± 0.11d	41.33 ± 2.45d	463.40 ± 310.99b	128.86 ± 80.17b	19.73 ± 6.85c	9.32 ± 3.42b
L1W	9.95 × 10^7^ ± 1.35	3.46 × 10^8^ ± 1.09	8.72 ± 0.01a	0.93 ± 0.02a	1.41 ± 0.03a	2.14 ± 0.24a	10.17 ± 0.56	14.33 ± 9.51a	0.51 ± 0.21a
L1C	2.69 × 10^7^ ± 1.10	8.66 × 10^7^ ± 1.54	8.97 ± 0.03b	2.38 ± 0.07a	1.84 ± 0.07a	3.44 ± 2.00a	16.46 ± 5.46	2.80 ± 0.97a	11.84 ± 2.79a
L2W	4.90 × 10^6^ ± 1.42	2.30 × 10^8^ ± 1.30	8.79 ± 0.01a	34.99 ± 0.13b	226.02 ± 1.61b	39.88 ± 7.27b	37.49 ± 13.98	3.76 ± 3.86a	0.97 ± 0.78b
L2C	4.56 × 10^5^ ± 1.25	5.01 × 10^7^ ± 1.11	9.56 ± 0.03c	14.81 ± 7.29c	85.28 ± 1.13c	89.04 ± 20.76c	42.03 ± 42.17	1.09 ± 0.65b	3.09 ± 1.78a
X1W	2.14 × 10^7^ ± 1.35	3.18 × 10^9^ ± 1.19	7.96 ± 0.03a	4.94 ± 0.06a	18.38 ± 0.19a	10.90 ± 0.20a	9.08 ± 0.54a	8.65 ± 0.75a	0.03 ± 0.01a
X1C	4.01 × 10^7^ ± 1.26	5.16 × 10^9^ ± 1.16	8.15 ± 0.02b	2.32 ± 0.17b	19.43 ± 1.25a	4.84 ± 0.32b	5.79 ± 0.59b	6.84 ± 0.28a	0.36 ± 0.01a
X2W	5.41 × 10^7^ ± 1.17	2.52 × 10^9^ ± 1.11	8.59 ± 0.02c	5.34 ± 0.04c	22.79 ± 0.18b	8.28 ± 0.09c	8.71 ± 0.67a	9.24 ± 0.54b	0.03 ± 0.01b
X2C	5.83 × 10^7^ ± 1.15	2.50 × 10^9^ ± 1.14	8.23 ± 0.03d	2.39 ± 0.05b	22.54 ± 0.22b	3.31 ± 0.36d	6.47 ± 0.77b	6.34 ± 0.81b	0.25 ± 0.04c

a P, Panlong Cave; X, Xincuntun Cave; L, Luohandu Cave; 1, at the middle of the cave; 2, at the end of the cave; W, weathered rock; C, weathered crust.

b USC, upland soil cluster.

c Different capital letters for values from one cave show significant differences (*P *< 0.05) among groups based on ANOVA.

Climate factors, such as the CH_4_ concentrations and air temperatures, showed similar spatial variation patterns across the three caves (Fig. 1C, Table S1 in the supplemental material). The CH_4_ concentrations decreased from the entrance inward to the caves (Fig. 1C), whereas temperatures showed a reverse pattern (Table S1). PLD had the lowest CH_4_ concentration (1.03 ± 0.02 ppm [mean ± standard deviation]) at the end of the cave (Fig. 1C, Table S1). However, the variations of atmospheric CO_2_ concentrations did not show a consistent pattern among the three caves (Fig. 1D).

### Diversity indices and microbial communities among the three karst caves.

Totals of 936,000 reads and 1,103,688 reads were obtained from *pmoA* gene and 16S rRNA gene amplicon sequencing, respectively, after quality control. The *pmoA* gene reads were clustered into 891 operational taxonomic units (OTUs) based on 95% similarity, whereas the 16S rRNA gene reads were grouped into 29,705 amplicon sequence variants (ASVs) with a 100% similarity cutoff.

**FIG 2:**
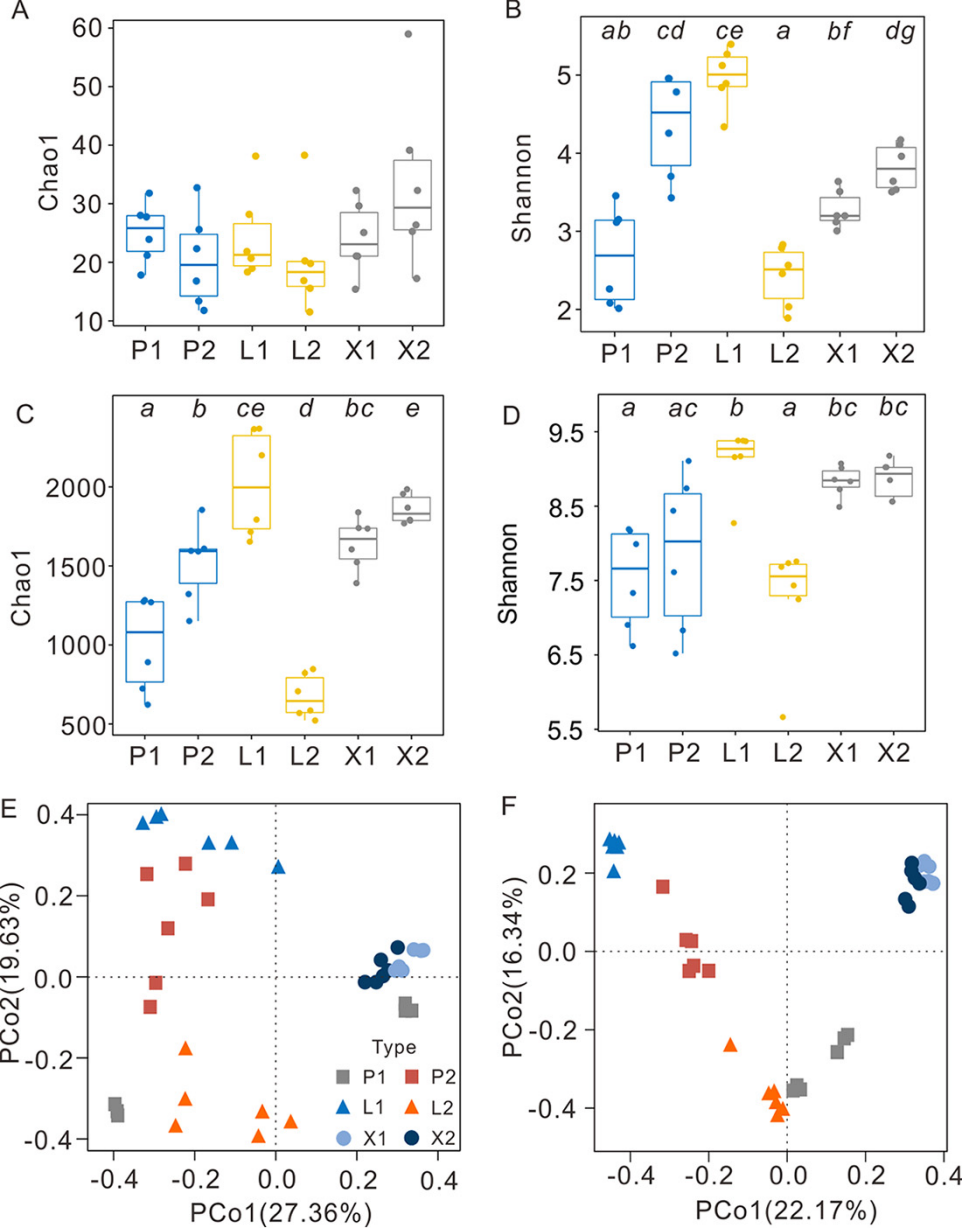
Alpha diversity and beta diversity indices of microbial communities in the three karst caves investigated in Guilin City, southwest China. (A to D) Chao1 and Shannon indices of atmMOB (atmospheric methane-oxidizing bacteria) (A, B) and the total bacterial communities (C, D) in weathered rock samples. Statistical significance, shown by different italic lowercase letters (*a* to *e*), was assessed by the Kruskal-Wallis *H* test (*P < *0.05). Principal coordinate analysis (PCoA) plots of the atmMOB (E) and bacterial (F) community structures. P, Panlong Cave; X, Xincuntun Cave; L, Luohandu Cave; 1, sample site at the middle of the cave; P2, sample site at the end of the cave.

Significant differences in alpha diversity indices in atmMOB and bacteria were observed in different cave samples (*P < *0.05) (Fig. 2A to D). The highest Shannon indices of both atmMOB and bacteria were observed in site L1 (at the middle of Luohandu Cave) samples, whereas the Shannon indices of site L2 (at the end of Luohandu Cave) samples were the lowest (Fig. 2B and D). The community structures of atmMOB and bacteria in XCT were significantly different from those in the other two caves (Fig. 2E and F). In the principal coordinate analysis (PCoA), the PCo1 and the PCo2 axis explained 27.36% and 19.63% of the variance in the atmMOB communities of all samples (Fig. 2E) and 22.17% and 16.34% of the variance in the total bacterial communities (Fig. 2F), respectively.

**FIG 3:**
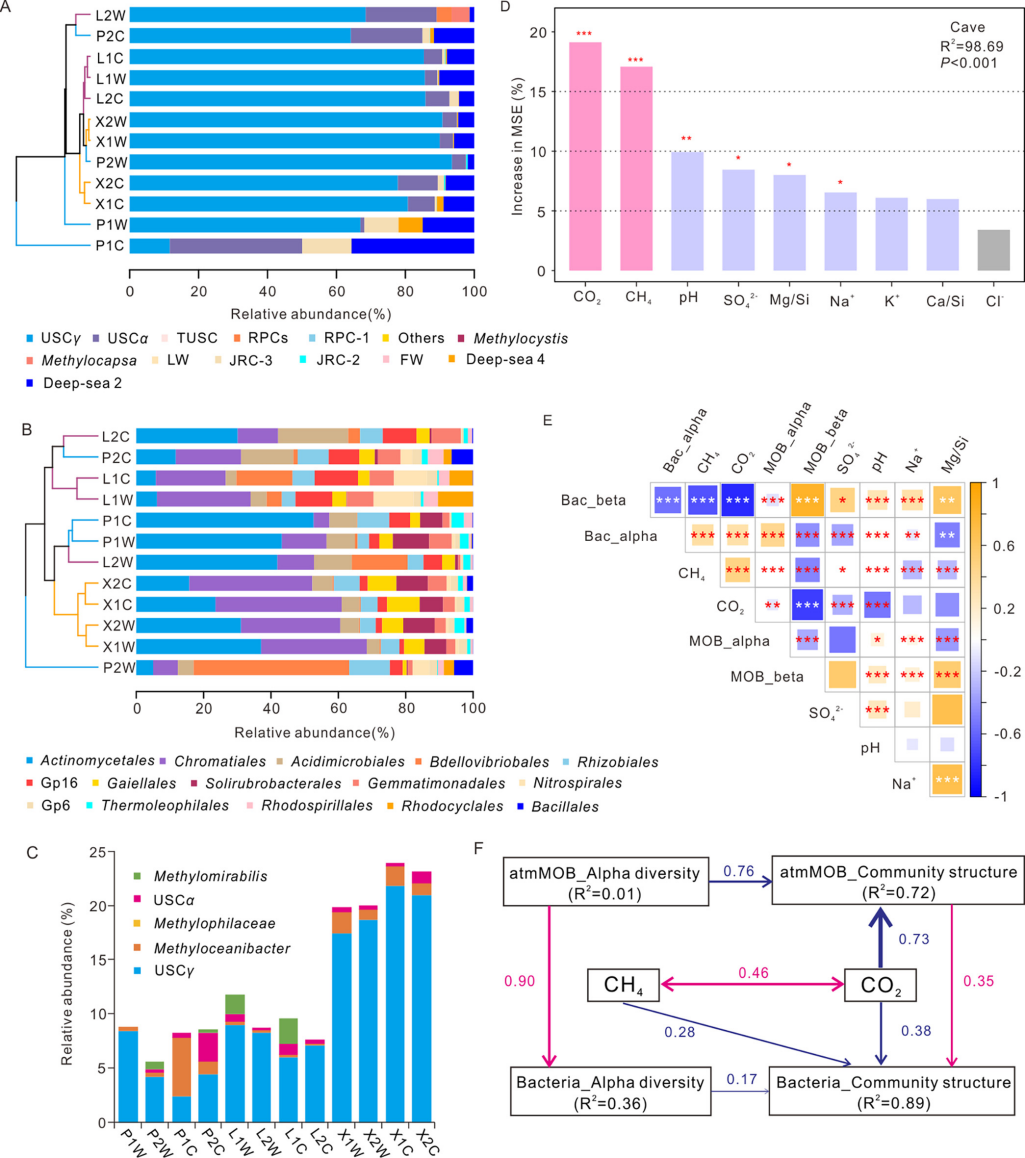
Microbial community structures and their correlation with environmental variables in the three karst caves in Guilin City, southwest China. Cluster analysis of the atmMOB (A) and total bacterial (B) community compositions in different niches of the three karst caves based on the Bray-Curtis distance and UPGMA method. The relative abundances of atmMOB clades (A) and bacterial orders (B) are shown using bar charts. USC, upland soil cluster; TUSC, tropical upland soil cluster; RPC, rice paddy cluster; LWs, Lake Washington sediments; JRC, Jasper Ridge cluster; FWs, freshwater sediment of Lake Wintergreen (27). (C) Genera of MOB as annotated based on 16S rRNA gene analysis from 36 samples. (D) Importance ranking (percentage of increase of mean square error [MSE]) of environmental variables as indicated by random forest machine learning. High MSE values mean more important predictors compared with low MSE values. (E) Correlation heatmap between important environmental variables and diversity indices in the three caves. The results of correlation analysis: *, 0.01 < *P < *0.05; **, 0.001 < *P < *0.01; ***, *P < *0.001. MOB_alpha and Bac_alpha represent the Shannon indexes of atmMOB and bacteria, respectively. (F) Structural equation model of atmospheric CH_4_ and CO_2_ interactions with atmMOB and total bacterial communities in the three karst caves. Solid arrows indicate significant effect sizes (*P* < 0.05), the thickness of arrows indicates the strength of the relationship, and red and blue indicate positive and negative relationships, respectively. P, Panlong Cave; X, Xincuntun Cave; L, Luohandu Cave; 1, sample site at the middle of the cave; 2, sample site far from the entrance of the cave; W, weathered rock; C, weathered crust.

USC*γ* dominated the atmMOB communities (>60%) in all caves, except for samples from P1C (weathered crust sampling point at the middle of Panlong Cave) (Fig. 3A). The USC*α* and Deep-sea 2 clades were the second and third most abundant groups of atmMOBs in all samples (Fig. 3A). Members of the rice paddy cluster (RPC) were relatively abundant in L2W (weathered rock sampling point at the end of Luohandu Cave), whereas members of the Deep-sea 2 clade and tropical upland soil cluster (TUSC) (27) were relatively abundant in P1C (Fig. 3A).

Quantification of USC*α* and USC*γ* using group-specific primers targeting the *pmoA* gene showed that USC*γ*, ranging from 10^5^ copies · g^−1 ^dry weight to 10^9^ copies · g^−1 ^dry weight, was more abundant than USC*α* (∼10^4^ to 10^7^ copies · g^−1 ^dry weight) in all samples (Table 1). The USC*γ* abundances in XCT were higher than those in LHD and PLD, whereas the highest abundance of USC*α* was observed in P2C (weathered crust sampling point at the end of Panlong Cave) (1.44 × 10^8^ ± 1.24 copies g^−1 ^dry weight) (Table 1).

For the total bacterial communities, *Actinobacteria* and *Proteobacteria* dominated in all samples at the phylum level (Table S2). Bacterial communities in XCT clustered together well, whereas bacterial communities in the PLD and LHD samples clustered according to sampling site (i.e., the middle or the end of the caves) (Fig. 3B). High proportions of unclassified bacterial taxa were observed in LHD (15.67% ± 5.31%), higher than those in PLD (9.07% ± 4.24%) and XCT (8.33 ± 0.60%) (Table S2). *Actinomycetales*, *Chromatiales*, and *Acidimicrobiales* were the most abundant orders in most weathered rock samples, except for P2W, in which *Bdellovibrionales* dominated (Fig. 3B). Within the phyla *Actinobacteria* and *Proteobacteria*, *Actinobacteria* and *Gammaproteobacteria* classes dominated in all weathered samples (Table S2). Among the three caves, USC accounted for 5.72% to 20.27% of the MOB communities, especially in XCT, accounting for 20.27% ± 2.19% (Fig. 3C, Table S2). XCT was also revealed to harbor the highest relative abundances of MOB, especially USC*γ*, as annotated from the known USC*γ* draft genome (ranging from 17% to 22%) (Fig. 3C). Moreover, *Methyloceanibacter* and USC*α* were also detected in some weathered rock samples (e.g., P1C and P2C) (Fig. 3C).

### Correlations between environmental parameters and microbial communities.

CO_2_ and CH_4_ concentrations, with high increases of mean square error (MSE) values, were the most important predictors in the total environmental parameters (*P < *0.001) as indicated by the random forest algorithm analysis (Fig. 3D). CH_4_ and CO_2_ concentrations were tightly linked with the community diversity and composition of atmMOB and bacteria in the karst caves (Fig. 3E). As shown by the result of structural equation model analysis, CO_2_ concentrations negatively affected the community structures of atmMOB (path coefficient = 0.73) and bacteria (path coefficient = 0.38). CH_4_ concentrations solely negatively influenced the community structures of bacterial communities (path coefficient = 0.28) (Fig. 3F). The diversity indices and community structures of atmMOB also had positive influences on those of bacteria (Fig. 3F). Moreover, the relative abundances of USC*γ* in atmMOB and bacterial communities connected positively with CH_4_ concentrations, whereas the relative abundances of USC*α* were negatively connected with CH_4_ concentrations across the three caves (Table S3). The atmMOB and total bacterial community compositions also correlated with other environmental parameters, such as pH and the concentrations of SO_4_^2−^ and Cl^−^ (Table 2). The *pmoA*-based relative abundances of USC*α* and Deep-sea 2 correlated positively with Cl^−^ and negatively with pH in all samples, whereas USC*γ* correlated negatively with the content of Cl^−^ and positively with pH (Table S3). The 16S rRNA-based relative abundances of *Methyloceanibacter* related negatively to pH, whereas *Methylomirabilis* related positively to pH (Table S3). The relative abundances of USC*γ* correlated negatively with Cl^−^ concentrations and positively with concentrations of CO_2_ and CH_4_ (Table S3). *Methyloceanibacter* relative abundances showed a positive correlation with Cl^−^ (Table S3).

**TABLE 2:** Mantel test results for the relationships between microbial communities and environmental factors in weathered rocks in karst caves in Guilin City, southwest China

Microbial community^*a*^	Cave^*b*^	Mantel test value (9,999 permutations)^*c*^:									
		All physicochemical parameters	Cl^−^	SO_4_^2−^	K^+^	Na^+^	pH	Ca/Si	Mg/Si	CH_4_	CO_2_
AtmMOB	PLD	0.43**	0.43**	0.80***	−0.02	−0.12	0.53**	0.29*	0.24	0.38**	0.38**
	LHD	0.71***	0.67***	0.69***	0.38*	0.31	0.004	−0.20	0.59**	0.46**	0.46**
	XCT	0.37**	0.60***	−0.07	0.62***	0.42**	0.37*	−0.04	0.10	0.02	0.02
	All caves	0.47***	0.51***	0.24*	0.24*	0.11	0.20**	0.27**	0.39***	0.35***	−0.06
Total bacteria	PLD	0.45**	0.05	0.71***	0.18	0.04	0.16	0.001	0.23	0.71**	0.71**
	LHD	0.79***	0.71***	0.74***	0.50**	0.29*	0.06	0.003	0.57***	0.86**	0.86**
	XCT	0.28*	0.43*	0.06	0.56**	0.28*	0.17	0.20	0.16	0.18	0.18
	All caves	0.15*	0.17*	0.36***	0.15	0.13	0.14*	0.14	0.17	0.27***	0.04

a AtmMOB, atmospheric methane-oxidizing bacteria.

b PLD, Panlong Cave; LHD, Luohandu Cave; XCT, Xincuntun Cave.

c Physicochemical parameters included all parameters in Table 1. Statistically significant results are in bold face: *, 0.01 < *P *< 0.05; **, 0.001< *P* < 0.01; ***, *P *< 0.001.

### Microbial cooccurrence networks.

**FIG 4:**
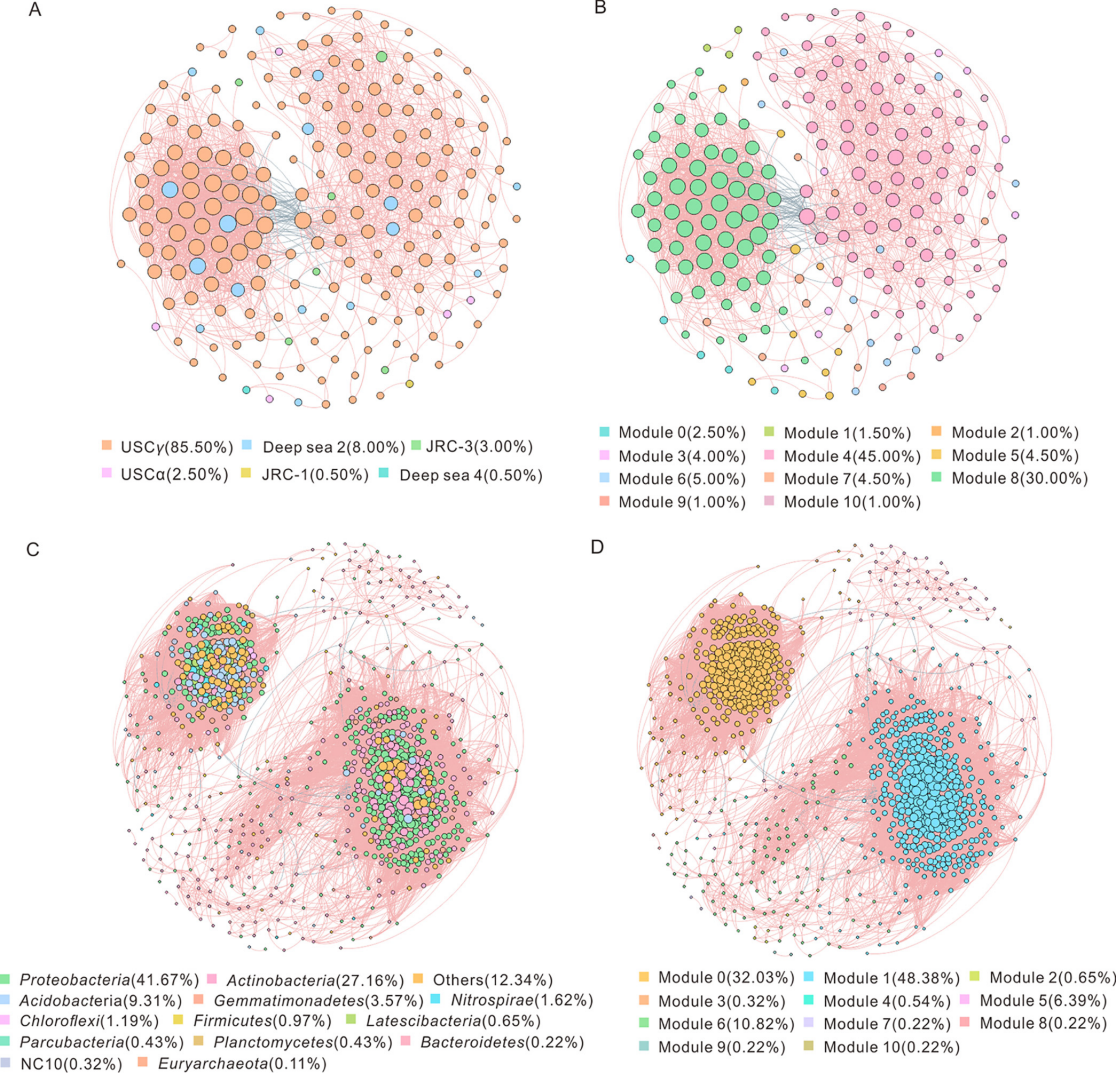
Cooccurrence networks of the atmMOB (A, B) and total bacterial (C, D) communities across three karst caves are colored to show taxonomy (A, C) and modules (B, D). Each node represents an OTU (atmMOB) or an ASV (total bacteria) in the network, and the node size is proportional to the degree (connected with other nodes). Nodes with positive interactions are linked with pink edges, whereas negative interactions are linked in blue.

The network of the atmMOB communities in all samples consisted of 200 nodes and 1,861 edges, and the network of bacteria was composed of 924 nodes and 58,418 edges (Table 3). The proportions of positive interactions were much higher (96.51% for atmMOB and 99.46% for bacteria) than those of negative interactions in both atmMOB and bacterial networks (Fig. 4). The modularity values of the two networks were slightly higher than 0.4 (Table 3), and there were 11 modules in the atmMOB network and 11 modules in the bacterial network (Fig. 4). Lower average path length (APL), higher average clustering coefficient (ACC), higher density, higher average degree (AD), and higher average weighted degree values were observed in the bacterial network than in the atmMOB network (Table 3).

**TABLE 3:** Topological properties of microbial cooccurrence networks in karst weathered rock, southwest China

Microbial network^*a*^	Value for^*b*^:								
	Nodes	Edges	APL	ACC	Diam	Modularity	Density	AD	AWD
AtmMOB	200	1,861	3.32	0.62	10	0.47	0.094	18.61	14.61
Total bacteria	924	58,418	3.28	0.73	9	0.49	0.14	126.45	101.94

a AtmMOB, atmospheric methane-oxidizing bacteria.

b APL, average path length; ACC, average clustering coefficient; Diam, diameter; AD, average degree; AWD, average weighted degree.

USC*γ* dominated in the atmMOB networks, accounting for 85.50% of the total nodes. The relative abundances of Deep-sea 2, JRC-3, USC*α*, JRC-1, and Deep-sea 4 in the atmMOB network were 8.00%, 3.00%, 2.50%, 0.50%, and 0.50%, respectively (Fig. 4A). In the bacterial network, *Proteobacteria* and *Actinobacteria* were the main phyla (Fig. 4C). Large modules (defined as modules with over 5% of the total nodes) in the network were associated with different caves (Table S4). *Latescibacteria* and NC10 (*Methylomirabilis*) were solely found in the LHD subnetwork, and *Bacteroidetes* was only found in the PLD subnetwork (Table S5).

**FIG 5:**
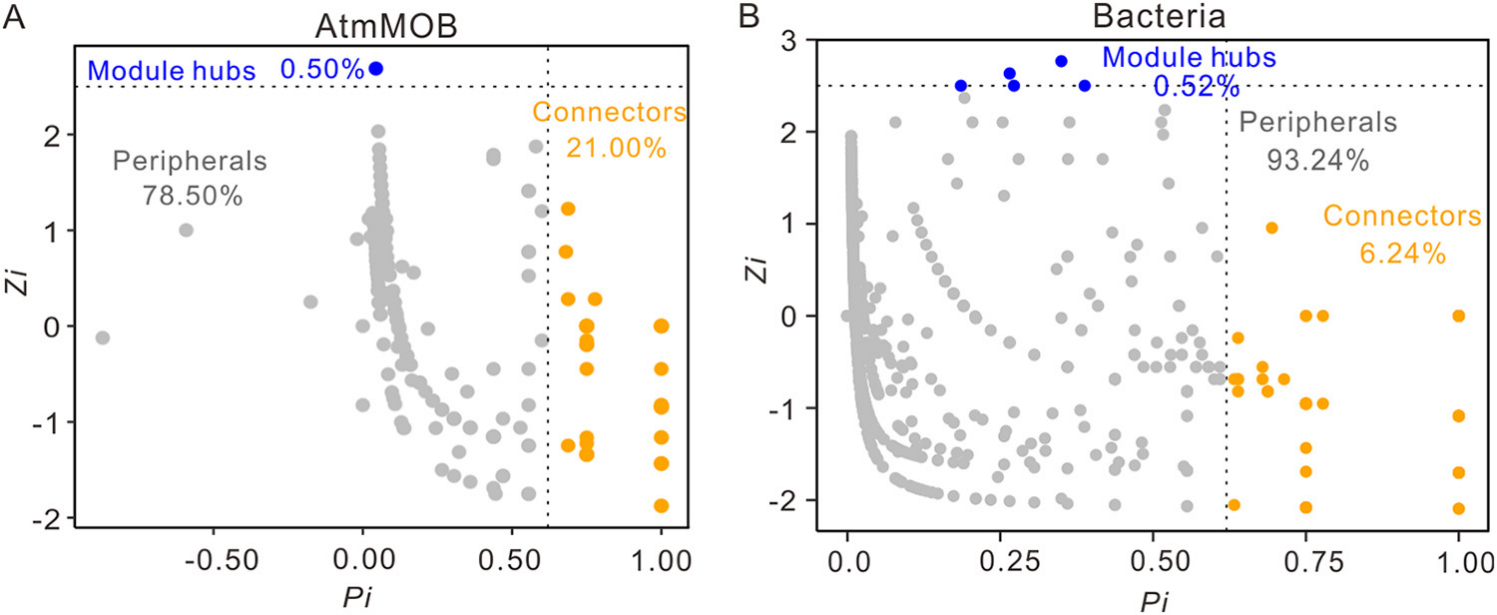
*Zi*-*Pi* plots showing the distribution of OTUs and ASVs with their topological roles in atmMOB (A) and bacterial (B) networks of three karst caves in Guilin City, southwest China. Each dot represents an OTU in the atmMOB network or an ASV in the bacterial network. *Zi*, within-module connectivity; *Pi*, among-module connectivity.

The results for the within-module connectivity (*Zi*)–among-module connectivity (*Pi*) relationships among OTUs and ASVs showed that in the atmMOB network most nodes (78.50%) were peripheral atmMOB, whereas 21.00% and 0.50% of the total OTU nodes were connectors and module hubs, respectively (Fig. 5A). In the bacterial network, 6.24% of the nodes were connectors and 0.52% were module hubs (Fig. 5B). No network hubs were found in the atmMOB or bacterial networks (Fig. 5). Connectors and module hubs were defined as keystone taxa (28, 29). USC*γ* was the major keystone taxon (accounting for 74.42%) in the atmMOB network (Fig. 5A, Table S6). In the bacterial network, 65 keystone taxa were observed, and the most abundant ones were affiliated with the phyla *Proteobacteria* (*Gammaproteobacteria* class and USC*γ* group) and *Actinobacteria* (*Gaiella* and *Aciditerrimonas*) (Fig. 5B, Table S7). In both atmMOB and bacterial networks, the relative abundances of keystone taxa all correlated positively with the CH_4_ and CO_2_ concentrations (Fig. S1). In the bacterial network, the MOB keystone taxon *Methyloceanibacter* solely accounted for 1.12% of all nodes, connected with a large number of other nodes (Fig. S2). USC*γ* was tightly connected with other bacterial nodes, including *Gaiella*, *Povalibacter*, *Bacillus*, and many other unclassified genera (Fig. S2).

## DISCUSSION

### The dominance of USC in the atmMOB communities in subterranean karst caves.

AtmMOB affiliated with USC were previously reported in various soils and were proposed to live by consumption of atmospheric CH_4_ (10, 14, 15). Therefore, soil has been considered the only biological sink of atmospheric CH_4_ in terrestrial ecosystems. Later, USC*α* was detected in biofilms of lava caves (12). Here, we revealed the dominance of USC in limestone and dolomite karst caves, which greatly expanded our understanding of the ecological distribution of USC in these ecosystems. Moreover, the high-throughput sequencing technique used in this study allows us to characterize the biodiversity of atmMOB in more detail. USC*α*, a lineage within the family *Beijerinckiaceae* (12, 17, 30), was mostly detected in cave ecosystems in biofilms and microbial mats from volcanic, limestone, and marble caves (12) and on weathered rocks in dolomite caves (Fig. 3A). It is worth noting that the relative abundances of USC*γ* assessed by *pmoA* gene sequencing were much higher than those of USC*α* in our samples (Fig. 3A), which was echoed by the results of 16S rRNA sequencing, especially in XCT (Fig. 3C). The results of the USC *pmoA* gene quantification showed intercave heterogeneity among the three karst caves, ranging from 10^4^ to 10^9^ copies · g^−1 ^dry weight (Table 1). The USC*α* abundances in these karst caves were between those of forest soil and grassland soil, whereas cave USC*γ* abundances were higher than those in forest and grassland soils (24). The dominance of USC*γ* may relate closely to alkaline conditions in our karst caves (Table 1) (16), conforming to our previous assumption. In addition to USC, we also observed additional minor MOB groups in the three karst caves investigated in this study, including Deep-sea 2, as suggested by *pmoA* gene sequencing (Fig. 3A), and *Methyloceanibacter* and *Methylomirabilis*, based on 16S rRNA sequencing (Fig. 3C). Of note, both Deep-sea 2 and *Methyloceanibacter* have been reported in anoxic sediments (18, 31), which were enriched in P1C samples in this study (Fig. 3A and C). Their occurrence may be highly related to the cave temperature (18.6°C) and pH (7.78 to 9.19), which might be favorable for these groups (18, 31). *Methyloceanibacter* has been isolated under conditions of 18 to 27°C and pH 6.3 to 9, with ammonium as the inorganic nitrogen source (18). Another anaerobic methanotrophic group detected was *Methylomirabilis* (i.e., NC10), which performs methane oxidation peculiarly coupled to denitrification. Site L1 harbored abundant *Methylomirabilis* (Fig. 3C), possibly due to the thick layer of weathered rock at this site, which may result in an anaerobic microenvironment.

### Environmental factors shape atmMOB and bacterial communities in karst caves.

pH and water gradients were the primary variables to shape MOB communities across large soil regions, whereas multiple variables, including the total nitrogen, aridity index, and mean annual temperature, affected the MOB community in small regions (11, 32). Correlations between environmental factors and MOB and total bacterial communities have been investigated in soils (11, 33), whereas such knowledge of karst caves is still scarce.

Our results showed that the pH and the CH_4_ concentration correlated significantly with atmMOB and other MOBs. Specifically, the relative abundances of USC*γ* and USC*α* had opposite associations with these environmental factors in the weathered rocks. The relative abundances of USC*γ* correlated positively but those of USC*α* correlated negatively with the CH_4_ concentration and pH (Table S3). The niche differentiation between USC*γ* and USC*α* according to pH has been well documented previously, showing that an alkaline pH favors USC*γ*, whereas neutral to slightly acidic conditions favor USC*α* (11, 24). The alkaline conditions observed in the cave samples may have resulted in the dominance of USC*γ*. The correlation between the relative abundance of USC and the pH further confirmed the different pH preferences of USC*α* and USC*γ.* Besides pH, we also found significant correlations between the relative abundance of USC and the CH_4_ concentration, as confirmed by the results of both *pmoA* sequencing and 16S rRNA sequencing in this study. Many studies have revealed that CH_4_ concentrations in karst caves are lower than in the outside atmosphere (3, 4). High CH_4_ concentrations were associated with increases in the USC*γ* relative abundances (Table S3), which might indicate that relatively high CH_4_ concentrations of close to 2 ppm are favorable for the growth of USC*γ*, especially in the X1 sampling site (Fig. 3A, Table S1). High relative abundances of USC*α* were observed under the low CH_4_ concentrations and neutral pHs at the P1C and P2C sites (Fig. 3A, Table S1), which provided suitable niches for USC*α*. These phenomena suggest that in addition to pH, the CH_4_ concentration may also drive the niche differentiation between USC*γ* and USC*α.* Excluding the CH_4_ concentration and pH, the CO_2_ concentration might also correlate positively with USC*γ* and negatively with USC*α* (Table S3). Type II MOB can fix CH_4_ and CO_2_ in the serine cycle (34). Recently, USC*γ* and USC*α* were both reported to contain genes for the serine cycle (16, 17), but USC*γ* might be more competitive with USC*α* in the cave environment of high CO_2_ concentrations and low [δ^13^C]CO_2_ value, especially in XCT (Table S1).

In addition to atmMOB, pH also affected the relative abundances of other MOBs, such as *Methyloceanibacter* and *Methylomirabilis*, based on the analysis of 16S rRNA sequencing (Table S3). The relative abundance of *Methyloceanibacter* linked negatively with pH, especially in P1C, which had the lowest pH (7.78 ± 0.01), whereas the relative abundance of *Methylomirabilis* correlated positively with pH and was rich in site L1 samples (pHs of 8.72 and 8.97). *Methylomirabilis* was mainly detected in the P2 and L1 sites, which had low CO_2_ concentrations, and the relative abundance of *Methylomirabilis* was revealed to be negatively correlated with CO_2_ (Table S3), suggesting that low CO_2_ concentrations were conducive to the subsistence of *Methylomirabilis*. Anaerobic MOB affiliated with *Methylomirabilis* have been reported to produce CO_2_ in the process of CH_4_ oxidization and to utilize CO_2_ in the Calvin-Benson-Bassham (CBB) cycle (35). This result suggested that anaerobic CH_4_ oxidization might decrease the CO_2_ concentration in anaerobic microenvironments of karst caves.

### Cooccurrence networks are dominated by positive links and USC*γ*.

Cooccurrence networks can serve as a powerful tool to investigate potential ecological interactions between microbial groups in natural environments, and network analysis may help to understand meaningful structural information of complex microbial taxa (36, 37). The cooccurrence network of atmMOB and bacteria showed mostly positive correlations (96.51% in the atmMOB network and 99.46% in the bacterial network) (Fig. 4), which indicated that members of the networks would respond simultaneously to environmental fluctuations, resulting in positive feedback and cooscillation (29, 38, 39). These phenomena suggested that MOB and bacteria were all susceptible to environmental changes.

The keystone taxa in the atmMOB and bacterial networks all belonged to module hubs and connectors (Fig. 5). USC*γ*, accounting for 85.50% of the total keystone taxa, predominated in the atmMOB network. It is worth noting that USC*γ* was also identified as a keystone taxon in the bacterial network (Table S7). USC*γ* is recalcitrant to culture and has no isolate to date, but a draft genome of the USC*γ* group has been obtained from alkaline mineral cryosols (16). The draft genome demonstrated that USC*γ* has all of the essential genes for the complete serine biosynthesis pathway (high-affinity type II MOB) for formaldehyde assimilation and genes involving nitrogen metabolism (16). Besides USC*γ*, USC*α* is the second keystone taxon in the atmMOB network. USCα may also be capable of nitrogen fixation, and it expresses the genes for hydrogenase and carbon monoxide dehydrogenase (17). Besides USC*γ*, the keystone taxa in the bacterial network also included *Gaiella* and *Aciditerrimonas* (Table S7). *Gaiella*, within the order *Gaiellales* in the phylum *Actinobacteria*, was first reported in a deep mineral aquifer (40). Functionally, *Gaiella* may be involved in the reduction of nitrate to nitrite (41, 42). *Aciditerrimonas* can live as both a heterotroph and an autotroph. Members of this genus are capable of ferric ion reduction to facilitate autotrophic growth under anaerobic conditions (43). Notably, *Aciditerrimonas* was reported in neutral soil (pH of 7.45 to 7.89), which correlated positively with total nitrogen (44). A subnetwork of keystone MOB nodes also showed that USC*γ* might connect with other bacteria, such as *Gaiella* and *Aciditerrimonas* (Fig. S2), which may be involved in the carbon and nitrogen cycles. In addition to USC*γ*, *Methyloceanibacter* connected with many nodes, especially USC*γ* and many unclassified nodes (Fig. S2). This result suggested that there might be a synergistic effect among MOBs to regulate the cave CH_4_ cycle. Collectively, these observations indicated that the keystone taxa in the atmMOB and bacterial occurrence network, especially USC, may be not only involved in the carbon cycle but also involved in or linked with the nitrogen cycle and other metabolic pathways. These findings offer us valuable information about the ecological relevance between elemental cycles in the caves.

### Conclusion.

In summary, wide distribution and dominance of high-affinity USC*γ* were observed for the MOB communities in subterranean karst caves, and caves offered more suitable habitats for USC*γ* than for USC*α*. Partially anoxic microniches in caves were also suitable for the growth of anaerobic MOB, especially *Methylomirabilis*. CH_4_ and CO_2_ concentrations, as the substrate and product of CH_4_ oxidation, respectively, and pH are key environmental factors affecting MOB community structure in caves. USC*γ* served as the keystone taxon both in the atmMOB and the overall bacterial cooccurrence networks, indicating the significance of this group in the total bacterial communities. The overwhelming dominance of positive links in the networks indicated a consistent response to environmental changes by different microbial groups and, thus, would have positive feedback in the cave ecosystem. In addition to participating in CH_4_ oxidation, USC in the weathered rock may also connect with multiple metabolic pathways, especially the nitrogen cycle. Our results greatly expand our knowledge about the ecological distribution of USC in natural environments and underline their significance in the consumption of atmospheric methane, supporting karst caves as another atmospheric methane sink besides soil in the terrestrial ecosystem.

## MATERIALS AND METHODS

### Study site description and sampling.

Guilin City in Guangxi Province is characterized by a well-developed and extensive distribution of karst physiognomy. The climate of this area is greatly influenced by subtropical monsoons, with a mean annual temperature of about 20°C and mean annual precipitation of around 1,887 mm (45, 46). Three distinct karst caves in Guilin City were selected for this study, which included Panlong Cave (PLD; 24°57′39.2″N, 110°21′17.4″E, with dripping water inside), Luohandu Cave (LHD; 25°0′55.8″N, 110°54′14.2″E, with subsurface stream and dripping water), and Xincuntun Cave (XCT; 24°58′38.5″N, 109°44′15.7″E, a dry cave without any water present during sampling) (Fig. 1A). The overlying vegetation varied from cave to cave. PLD was covered with shrubs, and the vegetation overlying LHD was dominated by arbors. In contrast, widely spaced orange trees were planted over XCT. The overlying strata of XCT were thinner (0.8 to 23 m) than those of the other two caves (PLD, 60 to 150 m; LHD, 3 to 136 m), and the well-developed cracks in the overlying rocks led to good ventilation at several sites inside the cave. The lengths of the three caves were 251 m for PLD, 356 m for LHD, and 100 m for XCT (Fig. 1C). PLD and XCT are limestone caves that developed in the Rongxian Formation of the Upper Devonian, and LHD is a dolomite cave developed in the Donggangling Formation of the Middle Devonian.

Samples were collected on 13 to 21 January 2019. At the approximate middle and the end of each cave, we sampled the weathered crust and the underlying weathered rocks on the cave wall using sterile spades. Triplicate samples of crust (C) and weathered rocks (W) were collected for each site (*n* = 36, contains 3 replicates). Air samples were collected with 1-liter gas sampling bags (MBT41; Dalian Hede Technologies Corporation, China). The air temperature was measured by an electronic thermometer (905-T1; Testo, Germany) while sampling. All solid samples were transported on ice to the geomicrobiology laboratory at China University of Geosciences (Wuhan) and stored at −80°C on arrival for further use.

### Physiochemical analysis.

All solid samples (*n* = 36, contains 3 replicates) were freeze-dried (Alpha 1-2 LD freeze-dryer; Martin Christ, Osterode am Harz, Germany) and passed through a sterile 2-mm sieve. The sieved samples were mixed with ultrapure water (1:5, wt/vol) to get a suspension. The supernatant pH of the suspension was determined using a multiparameter water quality detector (Hach, Loveland, CO, USA) (25). Dissolved anions and cations were measured with anionic chromatography (ICS-600; Thermo Scientific, USA) and inductively coupled plasma-optical emission spectrometry (ICP-OES) (iCAP 7600+; Thermo, USA), respectively, after filtration with 0.22-μm filters (47). The concentrations of CH_4_ and CO_2_ gases and the carbon isotope of CO_2_ ([δ^13^C]CO_2_) of cave air samples were measured by a high-precision carbon isotope analyzer (G2201-I; Picarro, USA) using cavity decay spectroscopy (cavity ring-down spectroscopy [CRDS]) (5) at the Institute of Karst Geology, Chinese Academy of Geological Sciences.

### DNA extraction, gene amplification, and sequencing.

An aliquot of 0.5 g of freeze-dried solid samples was used for DNA extraction with a DNeasy PowerSoil kit (12888-100; Qiagen, Germany) according to the manufacturer’s instructions. The concentration and quality of DNA were measured by a Nanodrop 2000 spectrophotometer (ND2000; Thermo Scientific, USA) and visualized by 2% agarose gel electrophoresis. Due to the dominance of USC*γ* in MOB via clone library construction with the primer set A189/mb661 in the Heshang Cave (22), the specific primer set A189f/A650r for the *pmoA* gene of atmMOB (21) and the 338F/806R primer set (48, 49) for bacterial V3-V4 16S rRNA were used, respectively. The resulting amplicons were sequenced on the Illumina MiSeq platform with a paired-end 250-bp (PE250) (for bacteria) and a PE300 (for *pmoA* gene) strategy at Shanghai Personal Biotechnology, Co., Ltd., Shanghai, China. All raw sequence reads were deposited in the National Omics Data Encyclopedia (NODE; https://www.biosino.org/node) with the project numbers OER094486 (for bacteria) and OER094488 (for MOB).

### *pmoA* gene quantification.

The absolute abundance of atmospheric methane-oxidizing bacteria (atmMOB) was measured by quantitative PCR (qPCR) to estimate the potency of atmMOB. The gene abundances of USC*γ* and USC*α* were determined using primer sets A189/gam634r (50) and A189/forest675r (51) and the TB Green system (RR820A; TaKaRa, Japan) with a real-time PCR detection system (CFX96; Bio-Rad, USA). All reactions were performed in triplicate in 20-μl volumes containing 1 μl template DNA, 10 μl 2× TB Green master mixture (RR820A; TaKaRa, Japan), 0.5 μl forward primer (10 μM), 0.5 μl reverse primer (10 μM), 3.2 μl 25 mM MgCl_2_, and 4.8 μl RNase-free water (H9012; TaKaRa, Japan). Standard curves were constructed with plasmid containing the target gene fragment, diluted to 10^9^ to 10^3^ gene copies · μl^−1^. The thermal cycling steps to determine USC gene abundance followed the protocols described previously in references 50 and 51, except that the annealing temperatures were 64°C for USC*α* and 64.5°C for USC*γ*. Triplicate PCRs were done for each of the triplicate environmental samples to quantify the *pmoA* gene abundances of the USC clades (*n* = 108, contains 9 replicates). The results of qPCR were expressed as gene copy numbers per gram dry weight (copies · g^−1^ dry weight). The average *R*^2^ of the standard curve was 0.997, and the amplification efficiency was within the range of 95% to 105%.

### Sequencing data processing.

For the *pmoA* and 16S rRNA genes, raw sequencing data were processed via the bcl2fastq software (version 1.8.4, Illumina) for primer cutting and barcode removal. The processed sequences were subsequently filtered and analyzed by QIIME 2 (Quantitative Insight Into Microbial Ecology; version 2019.7) (52). The sequence processing and removal of chimeric sequences of the *pmoA* gene were performed by VSEARCH (version 2.8.1) (53). The unique sequences were clustered at 95% sequence similarity to generate representative OTU sequences and an OTU table, and then all these 95%-level sequences were translated to amino acid sequences. The *pmoA* amino acid annotation was performed in BLAST 2.10.0+ (54) with an in-house-built database based on published data (11, 55, 56). 16S rRNA sequences were quality filtered with Q30, and chimeras were removed with the DADA2 plugin. Subsequently, representative amplicon sequence variant (ASV) sequences and a feature table were generated. Feature taxonomy of the 16S rRNA gene was assigned against a published database containing the sequences of atmMOB (10). All samples were resampled to the same level of sequencing to avoid the impact of sequencing depth on identifying microbial communities. Diversity indices included alpha diversity and beta diversity, and phylogenic trees (unrooted and rooted trees) were constructed in QIIME 2.

### Statistical analysis.

Spearman’s rho correlation, Pearson correlation, the Kruskal-Wallis *H* test, and analysis of variance (ANOVA) in SPSS Statistics (version 26.0) were used to investigate the correlations between environmental variables and communities and distinguish the differences in physicochemical parameters among different caves. Principal coordinate analysis (PCoA) and the Mantel test, both based on Bray-Curtis dissimilarities, were conducted with the vegan (57) package. The box plots of alpha diversity, linear relationship, and differential analysis of 16S rRNA and *pmoA* genes were analyzed and visualized with the ggpubr (58) and ggplot2 (59) packages of R software (version 3.6.1). Structural equation modeling was conducted with the AMOS (analysis of moment structures) software (version 25.0). Correlation heatmaps were analyzed and visualized with the corrplot package (60). The combination chart of stacked-bar and clustering tree-based unweighted pair group method using arithmetic average (UPGMA) was analyzed and visualized in R software. Random forest machine learning was performed with randomForest (61), A3 (62), and rfPermute (63) packages in R to explore the impacts of environmental variables in different caves.

Cooccurrence networks were constructed with Hmisc (64) and igraph (65) packages in R. To reduce the complexity, OTUs and ASVs that had relative abundances above 0.1% and more than 20% occurrence in all samples were selected for network analysis. Spearman’s correlation was calculated to explore the correlations among bacterial ASVs and atmMOB *pmoA* OTUs, with a correlation coefficient *ρ* of ≥0.7 and *P* value of <0.05 (Benjamini and Hochberg method adjusted). Networks were visualized with the Fruchterman-Reingold layout in Gephi (version 0.9.2) software (66). Within-module connectivity (*Zi*) and among-module connectivity (*Pi*) thresholds were used to classify the ecological roles of individual nodes in the network (67). Briefly, all nodes were classified into four groups: peripherals (*Zi *≤ 2.5 and *Pi *≤ 0.62), connectors (*Zi *≤ 2.5 and *Pi *> 0.62), module hubs (*Zi *> 2.5 and *Pi *≤ 0.62), and network hubs (*Zi *> 2.5 and *Pi *> 0.62) (68). Theoretically, connectors, module hubs, and network hubs were considered keystone taxa in the network (28, 69).
