# Supplementary material for: USCγ Dominated Community Composition and Cooccurrence Network of Methanotrophs and Bacteria in Subterranean Karst Caves
Source: Microbiol Spectr. 2021 Aug 18;9(1):10.1128/spectrum.00820-21. doi: 10.1128/spectrum.00820-21 (PMC8552738; doi:10.1128/spectrum.00820-21)
Supplement: SUPPLEMENTAL FILE 1 — Supplemental material. Download SPECTRUM00820-21_Supp_1_seq12.pdf, PDF file, 0.7 MB [file spectrum00820-21_supp_1_seq12.pdf]

Supporting Information

USCγ dominated community compositions and co-occurrence network of methanotrophs and bacteria in subterranean karst caves

Xiao-Yu Cheng[a,b†], Xiao-Yan Liu[a,b†], Hong-Mei Wang[a,b#], Chun-Tian Su[c], Rui Zhao[d], Paul L.E. Bodelier[e], Wei-Qi Wang[a,b], Li-Yuan Ma[a,b], Xiao-Lu Lu[a,b]

[a] State Key Laboratory of Biogeology and Environmental Geology, China University of Geosciences, Wuhan, 430074, China;

[b] School of Environmental Studies, China University of Geosciences, Wuhan 430074, China;

[c] Institute of Karst Geology, CAGS/Key Laboratory of Karst Dynamics, MNR&GZAR, Guilin 541004, China;

[d] School of Marine Science and Policy, University of Delaware, Lewes, DE 19958, USA;

[e] Department of Microbial Ecology, Netherlands Institute of Ecology (NIOO-KNAW), Droevendaalsesteeg 10, 6708 PB Wageningen, the Netherlands.

Corresponding author:

Hong-Mei Wang (wanghmei04@163.com or hmwang@cug.edu.cn)

[†] *Xiaoyu Cheng* and *Xiaoyan Liu* contributed equally to this work. Author order was determined by alphabetically.

Running title: Bacterial and methanotrophic communities in subsurface karst caves

Supplementary information

Fig. S1 Liner relationship between CH_4 and CO_2 concentrations and the relative abundance of keystone taxa based on the Bray-Curtis dissimilarity in MOB network (A, B) and bacterial network (C, D). Correlations between CH_4 and CO_2 concentrations and the Bray-Curtis dissimilarity of keystone taxa were analyzed via mantel test with Spearman's rho correlations.

Fig. S2 A sub-network of keystone MOB and other bacterial groups based on 16S rRNA in three karst caves, Guilin City. ASVs affiliated with keystone MOB in bacterial occurrence network and those with a significant correlation with keystone MOB ASVs were selected for the network analysis. Each node represents an ASV in the bacterial network, and the size of node is proportional to degree (connected with other nodes). Individual nodes with positive interactions are linked with pink edges, whereas negative interactions are in gray.

Table S1 The concentrations of CH_4 and CO_2, temperature in three karst caves in Guilin City, southwestern China

Table S2 Bacterial compositions at different taxonomic levels in the three karst caves investigated in Guilin city, southwestern China

Table S3 Pearson correlations between the relative abundance of major atmMOB and bacterial groups and physicochemical properties in three karst caves in Guilin city, southwestern China

Table S4 Relative abundance of MOB OTUs and bacterial ASVs within individual modules in the sub-networks of three karst caves in Guilin City, southwestern China

Table S5 The relative abundance of bacterial phyla in sub-networks of three karst caves and the total network across the three caves in Guilin City, southwestern China

Table S6 Topological indices of keystone taxa in the MOB network in Guilin city, southwestern China

Table S7 Topological indices of keystone taxa in the bacterial network of three caves in Guilin city, southwestern China

Table S8 The potential methane oxidation rate estimated in the weathered rock samples in three karst caves in Guilin city, southwestern China

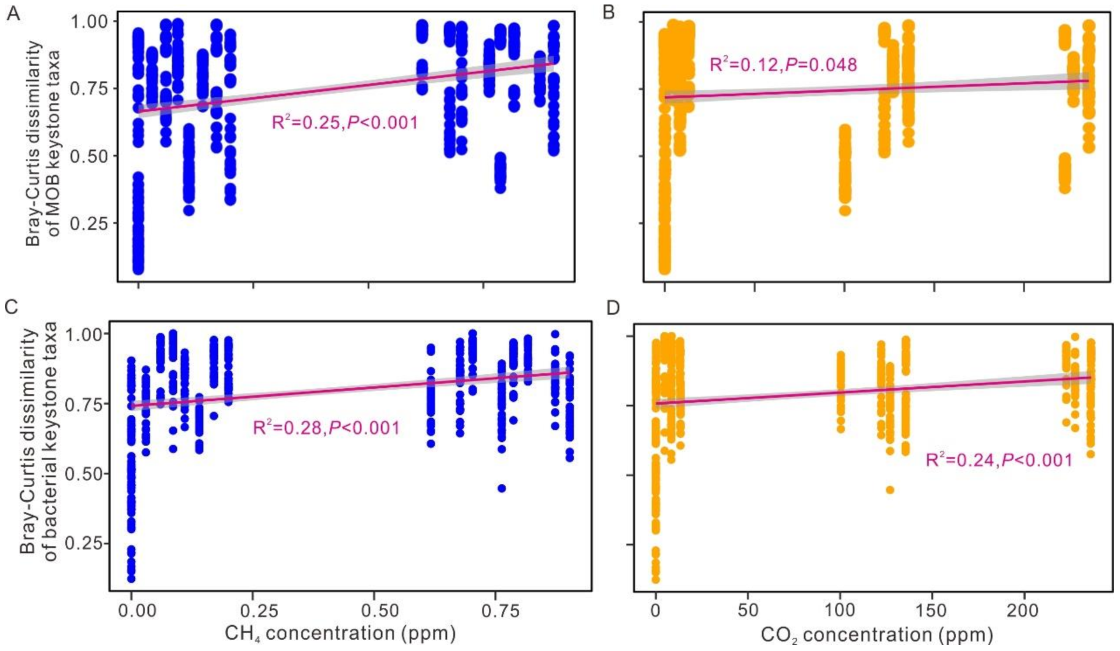

Fig. S1 Liner relationship between CH_4 and CO_2 concentrations and the relative abundance of keystone taxa based on the Bray-Curtis dissimilarity in MOB network (A, B) and bacterial network (C, D). Correlations between CH_4 and CO_2 concentrations and the Bray-Curtis dissimilarity of keystone taxa were analyzed via mantel test with Spearman's rho correlations.

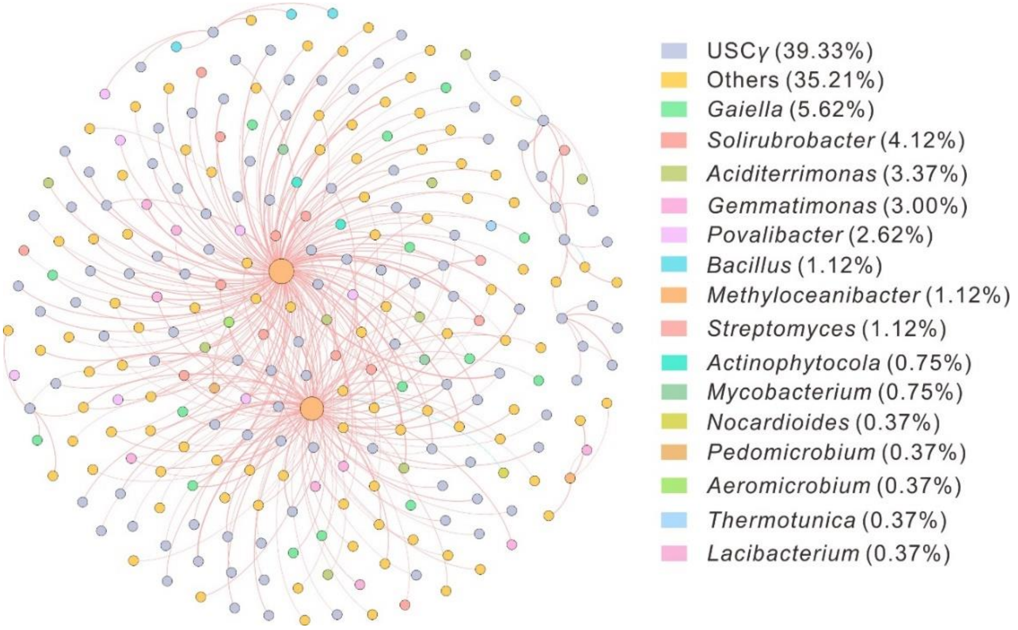

Fig. S2 A sub-network of keystone MOB and other bacterial groups based on 16S rRNA in three karst caves, Guilin City. ASVs affiliated with keystone MOB in bacterial occurrence network and those with a significant correlation with keystone MOB ASVs were selected for the network analysis. Each node represents an ASV in the bacterial network, and the size of node is proportional to degree (connected with other nodes). Individual nodes with positive interactions are linked with pink edges, whereas negative interactions are in gray.

Table S1 The concentrations of CH_4 and CO_2, temperature in three karst caves in Guilin City, southwestern China

Sample ID	CH_4 (ppm)	CO_2 (ppm)	Temperature (°C)	$\delta^{13}C$-CO_2 (‰)
P1	1.11 ± 0.02	474.7 ± 0.28	18.6	-13.80 ± 0.13
P2	1.03 ± 0.02	469.9 ± 0.36	19.3	-13.72 ± 0.16
L1	1.93 ± 0.02	461.5 ± 0.27	14.0	-16.51 ± 0.36
L2	1.73 ± 0.02	461.3 ± 0.40	17.4	-16.48 ± 0.15
X1	1.90 ± 0.01	697.4 ± 0.19	15.5	-18.65 ± 0.02
X2	1.79 ± 0.02	597.0 ± 0.19	17.3	-17.42 ± 0.05

P1: near the entrance of Panlong Cave. P2: at the end of Panlong Cave. The same nomenclature was applied for all samples in the three caves. L: Luohandu Cave; X: Xincuntun Cave.

Table S2 Bacterial compositions at different taxonomic levels in the three karst caves investigated in Guilin city, southwestern China

Taxonomic units	Taxonomy	PLD	LHD	XCT
	Actinobacteria	39.01 ± 22.90%	29.46 ± 21.96%	42.19 ± 4.39%
	Proteobacteria	34.00 ± 15.63%	31.19 ± 5.83%	37.70 ± 2.49%
	Unclassified	9.07 ± 4.24%[ab]	15.67 ± 5.31%[a]	8.33 ± 0.60%[b]
	Acidobacteria	8.77 ± 2.92%	12.63 ± 5.71%	6.13 ± 0.82%
	Gemmatimonadetes	2.88 ± 1.73%	3.41 ± 1.86%	2.81 ± 0.70%
Phylum	*Firmicutes*	2.01 ± 1.81%	0.36 ± 0.15%	0.76 ± 0.70%
	Nitrospirae	1.53 ± 1.26%	2.68 ± 2.61%	0.65 ± 0.19%
	Planctomycetes	0.62 ± 0.37%	0.55 ± 0.50%	0.83 ± 0.14%
	Parcubacteria	0.39 ± 0.15%	0.42 ± 0.41%	0.19 ± 0.11%
	Latescibacteria	0.37 ± 0.30%	0.69 ± 0.70%	0.06 ± 0.03%
	Bacteroidetes	0.36 ± 0.45%	0.13 ± 0.08%	0.08 ± 0.03%
	Chloroflexi	0.35 ± 0.28%	1.38 ± 1.22%	0.11 ± 0.05%
	NC10	0.26 ± 0.30%	1.03 ± 1.04%	/
	Aenigmarchaeota	0.09 ± 0.05%[a]	0.01 ± 0.01%[b]	0.03 ± 0.02%[b]
	Verrucomicrobia	0.07 ± 0.05%	0.03 ± 0.04%	0.10 ± 0.12%
	Actinobacteria	35.61 ± 20.20%	28.21 ± 21.04%	37.59 ± 4.86%
	Gammaproteobacteria	12.07 ± 2.17%[a]	13.31 ± 2.70%[a]	29.93 ± 2.15%[b]
	Betaproteobacteria	9.76 ± 14.14%	9.77 ± 4.07%	1.47 ±1.15%
Order	*Alphaproteobacteria*	9.31 ± 3.57%	4.93 ± 0.42%	4.98 ± 0.86%
	Deltaproteobacteria	2.56 ± 1.02%	2.92 ± 2.34%	1.04 ± 0.25%
	Thermoleophilia	1.50 ± 1.01%	0.78 ± 0.56%	1.32 ± 0.55%
	Oligoflexia	0.003 ± 0.004%	/	/
	USCγ	4.86 ± 2.77%[a]	7.59 ± 3.06%[b]	19.70 ± 2.10%[c]
	Methyloceanibacter	1.89 ± 2.67%[a]	0.32 ± 0.16%[b]	1.53 ± 0.47%[ab]
Genus	USCα	0.86 ± 1.09%	0.58 ± 0.35%	0.58 ± 0.31%
	Methylomirabilis	0.26 ± 0.39%[a]	1.03 ± 1.09%[b]	/
	Methylococcaceae	0.004 ± 0.006%	/	0.0003 ± 0.0005%
	Methylophilaceae	0.001 ± 0.004%	/	/

Values with the same letter are not different via ANOVA analyses ($P < 0.05$). PLD: Panlong Cave, LHD: Luohandu Cave, XCT: Xincuntun Cave. /: not detected

Table S3 Pearson correlations between the relative abundance of major atmMOB and bacterial groups and physicochemical properties in three karst caves in Guilin city, southwestern China.

Target microbial communities	Group	Cl^-	SO_4^{2-}	K^+	Na^+	pH	Ca/Si	Mg/Si	CH_4	CO_2
	USC*γ*	**-0.69****	-0.08	-0.11	-0.06	**0.44****	**-0.71****	**-0.37***	**0.47****	0.26
atmMOB based on	Deep-sea 2	**0.43****	-0.32	0.08	0.01	**-0.48****	**0.78****	0.15	**-0.43****	-0.16
pmoA	JRC-3	**0.43****	-0.13	-0.07	-0.07	-0.18	**0.58****	0.13	**-0.56****	-0.28
	Deep-sea 4	-0.24	-0.19	0.01	-0.03	0.21	-0.16	-0.13	-0.32	-0.04
	USC*α*	**0.80****	0.28	0.20	0.17	**-0.49****	**0.67****	**0.51****	**-0.36***	-0.23
	Actinobacteria	**0.47****	**0.37***	-0.11	-0.08	0.16	0.33	-0.19	0.18	-0.12
	Gammaproteobacteria	**-0.45****	-0.33	-0.18	-0.24	-0.13	**-0.41***	**-0.48****	**0.90****	**0.53****
	Betaproteobacteria	-0.10	0.21	-0.11	-0.11	-0.29	-0.12	**0.42***	**-0.33***	-0.19
	Alphaproteobacteria	**0.46****	-0.03	0.23	0.18	**0.53****	0.21	-0.27	-0.28	**-0.62****
	Deltaproteobacteria	**-0.38***	**-0.43****	0.14	0.11	-0.02	-0.20	0.29	**-0.46****	-0.06
Bacteria based on	*Thermoleophilia*	**0.46****	0.14	-0.11	-0.13	**0.40***	0.23	**-0.33***	0.05	-0.26
16S rRNA	*Oligoflexia*	0.19	0.01	**0.51****	**0.48****	0.21	0.24	-0.08	-0.21	**-0.50****
	USC*γ*	**-0.49****	-0.23	-0.27	-0.29	-0.31	-0.26	**-0.40***	**0.59****	**0.81****
	Methyloceanibacter	**0.43****	-0.16	-0.03	-0.07	**-0.58****	**0.65****	0.11	-0.23	0.14
	USC*α*	0.22	-0.09	**0.59****	**0.53****	-0.27	0.29	0.29	-0.24	-0.12
	Methylomirabilis	-0.10	0.03	0.07	0.11	**0.51****	-0.08	-0.07	0.11	**-0.38***
	Methylococcaceae	-0.13	0.04	-0.08	-0.10	0.27	-0.14	-0.15	**-0.34***	-0.14

Methylophilaceae	-0.09	0.03	-0.06	-0.06	0.21	-0.12	-0.11	-0.25	-0.11

The results with 0.01< $P < 0.05$ and $P < 0.01$ were respectively marked with * and ** and bolded. PLD: Panlong Cave; LHD: Luohandu Cave; XCT: Xincuntun Cave.

Table S4 Relative abundance of MOB OTUs and bacterial ASVs within individual modules in the sub-networks of three karst caves in Guilin City, southwestern China

MOB Modules	PLD	LHD	XCT	Bacterial Modules	PLD	LHD	XCT
Module 0 (2.50%)	32.38%	3.31%	**64.41%**	**Module 0 (32.03%)**	35.97%	**54.08%**	9.95%
Module 1 (1.50%)	0.12%	**99.18%**	0.70%	**Module 1 (48.38%)**	16.59%	12.31%	**71.10%**
Module 2 (1.00%)	**61.36%**	20.52%	18.12%	Module 2 (0.65%)	**69.64%**	30.36%	0
Module 3 (4.00%)	31.91%	**40.33%**	27.77%	Module 3 (0.32%)	**57.14%**	37.24%	5.61%
**Module 4 (45.00%)**	37.49%	**58.82%**	3.70%	Module 4 (0.54%)	**55.49%**	1.70%	42.80%
Module 5 (4.50%)	25.06%	28.13%	**46.82%**	**Module 5 (6.39%)**	36.99%	**48.22%**	14.79%
Module 6 (5.00%)	**90.08%**	5.11%	4.80%	**Module 6 (10.82%)**	**56.58%**	11.12%	32.30%
Module 7 (4.50%)	23.56%	16.07%	**60.37%**	Module 7 (0.22%)	35.16%	16.02%	48.83%
**Module 8 (30.00%)**	4.27%	2.67%	**93.06%**	Module 8 (0.22%)	25.66%	9.39%	64.96%
Module 9 (1.00%)	**48.33%**	19.54%	32.12%	Module 9 (0.22%)	24.68%	0.54%	74.77%
Module 10 (1.00%)	**91.88%**	1.13%	6.99%	Module 10 (0.22%)	10.64%	41.97%	47.39%

Percentage numbers in the parenthesis refer to the percentage of the nodes in individual modules to the total nodes of the network. Bolded modules are large modules with a node number over 5% of the total nodes. The highest relative abundances of MOB OTUs and bacterial ASVs in the three caves were bolded. PLD: Panlong Cave; LHD: Luohandu Cave; XCT: Xincuntun Cave.

Table S5 The relative abundance of bacterial phyla in sub-networks of three karst caves and the total network across the three caves in Guilin City

Taxonomy of nodes	Relative abundance (%)			
	PLD	LHD	XCT	Whole
Proteobacteria	23.68	31.83	53.85	41.67
Planctomycetes	0.88	0.85	/	0.43
Parcubacteria	0.88	0.28	0.44	0.43
Others	7.02	19.44	8.13	12.34
Nitrospirae	1.75	3.38	0.22	1.62
Latescibacteria	/	1.69	/	0.65
Gemmatimonadetes	2.63	2.82	4.40	3.57
Firmicutes	2.63	1.13	0.44	0.97
Euryarchaeota	/	0.28	/	0.11
Chloroflexi	/	2.82	0.22	1.19
Candidate division NC10	/	0.85	/	0.32
Bacteroidetes	1.75	/	/	0.22
Actinobacteria	55.26	16.06	28.79	27.16
Acidobacteria	3.51	18.59	3.52	9.31

PLD:Panlong Cave; LHD: Luohandu Cave; XCT: Xincuntun Cave.

Table S6 Topological indices of keystone taxa in the MOB network in Guilin city, southwestern China

Group	No. Node	Node information	Within module connectivities (*Zi*)	Among module connectivities (*Pi*)	Degree	Closness Centrality	Betweeness Centrality	Modularity class
Module hub	OTU_5639	g_USC*γ*	2.69	0.04	45	0.41	1139.38	4
Connector	OTU_10	g_USC*γ*	0	1	1	1.00	0	10
	OTU_15959	g_USC*γ*	0	1	1	1.00	0	10
	OTU_427	g_USC*γ*	0	1	1	0.18	0	9
	OTU_50	g_JRC3	0	1	1	1.00	0	2
	OTU_57	g_JRC3	0	1	1	1.00	0	2
	OTU_153	g_USC*α*	-0.45	1	1	0.17	0	0
	OTU_15247	g_USC*γ*	-0.83	1	1	0.25	0	7
	OTU_2711	g_USC*γ*	-0.83	1	1	0.25	0	7
	OTU_102	g_USC*γ*	-0.85	1	1	0.19	0	3
	OTU_15100	g_USC*γ*	-0.85	1	1	0.17	0	3
	OTU_171	g_USC*γ*	-0.85	1	1	0.17	0	3
	OTU_6489	g_USC*γ*	-0.85	1	1	0.20	0	3

OTU_7	g_Deep-sea 2	-1.16	1	1	0.20	0	6
OTU_77	g_USCγ	-1.16	1	1	0.16	0	6
OTU_4064	g_USCγ	-1.44	1	1	0.25	0	4
OTU_5	g_USCγ	-1.44	1	1	0.23	0	4
OTU_53	g_USCγ	-1.44	1	1	0.24	0	4
OTU_7553	g_USCγ	-1.44	1	1	0.26	0	4
OTU_2731	g_Deep-sea 2	-1.88	1	1	0.26	0	8
OTU_6097	g_USCγ	-1.88	1	1	0.24	0	5
OTU_4876	g_USCγ	0.28	0.78	3	0.25	782.87	3
OTU_11	g_USCγ	0	0.75	2	1.00	0	1
OTU_170	g_JRC1	0	0.75	2	0.23	191.00	9
OTU_32	g_Deep-sea 2	0	0.75	2	0.21	0	5
OTU_6	g_USCγ	0	0.75	2	1.00	0	1
OTU_78	g_Deep-sea 4	0	0.75	2	0.21	0	5
OTU_82	g_USCγ	0	0.75	2	1.00	0	1
OTU_9689	g_JRC3	-0.15	0.75	2	0.23	0	7
OTU_14	g_JRC3	-0.19	0.75	2	0.17	0	6

OTU_2	g_USCγ	-0.19	0.75	2	0.22	370.40	6
OTU_230	g_USCα	-0.19	0.75	2	0.20	0	6
OTU_19	g_USCγ	-0.45	0.75	2	0.25	76.64	0
OTU_114	g_USCγ	-1.16	0.75	2	0.28	548.13	6
OTU_4009	g_USCγ	-1.22	0.75	2	0.26	213.75	5
OTU_11275	g_USCγ	-1.34	0.75	2	0.26	0	4
OTU_1436	g_USCγ	-1.34	0.75	2	0.27	0.50	4
OTU_411	g_USCγ	-1.34	0.75	2	0.25	0	4
OTU_46	g_USCγ	-1.34	0.75	2	0.27	0	4
OTU_37	g_USCγ	1.22	0.69	4	0.26	440.63	5
OTU_27525	g_USCγ	0.28	0.69	4	0.25	438.75	3
OTU_122	g_USCγ	-1.25	0.69	4	0.31	409.89	4
OTU_45	g_USCγ	0.77	0.68	5	0.26	925.24	6

Table S7 Topological indices of keystone taxa in the bacterial network of three caves in Guilin city, southwestern China.

Group	No. Node	Node information	Within module connectivities (*Zi*)	Among module connectivities (*Pi*)	Degree	Closness Centrality	Betweeness Centrality	Modularity class
Module hub	ASV_12142	p_ *Actinobacteria*; c_ *Actinobacteria*; o_ *Acidimicrobiales*; f_ *Acidimicrobineae incertae sedis*; g_ *Aciditerrimonas*	2.50	0.27	32	0.28	651.56	6
	ASV_15837	p_ *Actinobacteria*; c_ *Actinobacteria*; o_ *Solirubrobacterales*	2.77	0.35	37	0.29	1227.20	6
	ASV_22963	p_ Others	2.63	0.27	33	0.28	625.56	6
	ASV_3158	p_ *Proteobacteria*; c_ *Alphaproteobacteria*; o_ *Rhizobiales*	2.50	0.19	30	0.28	556.11	6
	ASV_641	p_ *Proteobacteria*; c_ *Alphaproteobacteria*; o_ *Rhizobiales*; f_ *Hyphomicrobiaceae*; g_ *Hyphomicrobium*	2.50	0.39	36	0.29	707.83	6
Connector	ASV_10382	p_ *Actinobacteria*; c_ *Actinobacteria*	-2.08	0.75	2	0.22	0	0
	ASV_10590	p_ *Actinobacteria*; c_ *Actinobacteria*; o_ *Actinomycetales*; f_ *Mycobacteriaceae*; g_ *Mycobacterium*	-0.95	0.75	2	0.20	591.62	6
	ASV_10682	p_ *Actinobacteria*; c_ *Actinobacteria*; o_ *Acidimicrobiales*; f_ *Acidimicrobineae_incertae_sedis*; g_ *Aciditerrimonas*	-1.09	1	1	0.24	0	6
	ASV_10836	p_ *Actinobacteria*; c_ *Actinobacteria*; o_	-0.95	0.75	2	0.21	0	5

	Gaiellales; f_ *Gaiellaceae*; g_ *Gaiella*						
ASV_11184	p_ *Actinobacteria*; c_ *Actinobacteria*; o_ *Actinomycetales*; f_ *Pseudonocardiaceae*	-1.09	1	1	0.20	0	5
ASV_11793	p_ *Actinobacteria*; c_ *Actinobacteria*; o_ *Acidimicrobiales*; f_ *Acidimicrobineae_incertae_sedis*; g_ *Aciditerrimonas*	-1.09	1	1	0.17	0	5
ASV_11982	p_ *Actinobacteria*; c_ *Actinobacteria*; o_ *Acidimicrobiales*; f_ *Acidimicrobineae_incertae_sedis*; g_ *Aciditerrimonas*	-0.95	0.75	2	0.23	0	5
ASV_12022	p_ *Actinobacteria*; c_ *Actinobacteria*; o_ *Actinomycetales*	-1.70	1	1	0.23	0	1
ASV_12076	p_ *Actinobacteria*; c_ *Actinobacteria*; o_ *Acidimicrobiales*; f_ *Acidimicrobineae_incertae_sedis*; g_ *Aciditerrimonas*	0	1	1	1	0	8
ASV_12115	p_ *Actinobacteria*; c_ *Thermoleophilia*; o_ *Thermoleophilales*; f_ *Thermoleophilaceae*; g_ *Thermoleophilum*	-0.95	0.75	2	0.23	0	6
ASV_12250	p_ *Actinobacteria*; c_ *Actinobacteria*; o_ *Solirubrobacterales*; f_ *Solirubrobacteraceae*; g_ *Solirubrobacter*	-1.09	1	1	0.20	0	6
ASV_12363	p_ *Actinobacteria*; c_ *Actinobacteria*; o_ *Gaiellales*; f_ *Gaiellaceae*; g_ *Gaiella*	0	1	1	1	0	7
ASV_12541	p_ *Actinobacteria*; c_ *Actinobacteria*; o_	-1.09	1	1	0.20	0	6

	Solirubrobacterales; f_ *Solirubrobacteraceae*; g_ *Solirubrobacter*						
ASV_12631	p_ *Actinobacteria*; c_ *Actinobacteria*	-0.82	0.69	4	0.26	5.32	6
ASV_12940	p_ *Actinobacteria*; c_ *Actinobacteria*; o_ *Actinomycetales*; f_ *Pseudonocardiaceae*; g_ *Actinophytocola*	-0.95	0.75	2	0.23	34.32	6
ASV_13197	p_ *Actinobacteria*; c_ *Actinobacteria*; o_ *Acidimicrobiales*; f_ *Acidimicrobineae_incertae_sedis*; g_ *Aciditerrimonas*	-2.10	1	1	0.22	0	0
ASV_13739	p_ *Actinobacteria*; c_ *Actinobacteria*; o_ *Gaiellales*; f_ *Gaiellaceae*; g_ *Gaiella*	-1.09	1	1	0.21	0	5
ASV_14084	p_ *Actinobacteria*; c_ *Actinobacteria*; o_ *Actinomycetales*; f_ *Pseudonocardiaceae*	-0.95	0.75	2	0.21	0	5
ASV_14674	p_ *Actinobacteria*; c_ *Actinobacteria*; o_ *Gaiellales*; f_ *Gaiellaceae*; g_ *Gaiella*	0	1	1	1	0	7
ASV_14767	p_ *Actinobacteria*; c_ *Actinobacteria*; o_ *Acidimicrobiales*; f_ *Acidimicrobineae_incertae_sedis*; g_ *Aciditerrimonas*	0	1	1	1	0	8
ASV_14915	p_ *Actinobacteria*; c_ *Actinobacteria*; o_ *Acidimicrobiales*; f_ *Acidimicrobineae_incertae_sedis*; g_ *Aciditerrimonas*	-1.09	1	1	0.20	0	6
ASV_14944	p_ *Actinobacteria*; c_ *Actinobacteria*; o_ *Gaiellales*; f_ *Gaiellaceae*; g_ *Gaiella*	-0.82	0.69	4	0.29	952.12	5

ASV_15078	p_ *Actinobacteria*; c_ *Actinobacteria*; o_ *Acidimicrobiales*; f_ *Acidimicrobiaceae*; g_ *Ilumatobacter*	-0.95	0.75	2	0.17	35.35	6
ASV_15290	p_ *Actinobacteria*; c_ *Actinobacteria*; o_ *Gaiellales*; f_ *Gaiellaceae*; g_ *Gaiella*	-0.95	0.75	2	0.20	546.07	5
ASV_15681	p_ *Actinobacteria*; c_ *Actinobacteria*; o_ *Actinomycetales*	-0.95	0.78	3	0.25	998.06	5
ASV_17013	p_ *Acidobacteria*	-2.08	0.75	2	0.18	0	0
ASV_1706	p_ *Proteobacteria*; c_ *Gammaproteobacteria*; o_ *Chromatiales*; f_ unclassified_*Chromatiales*; g_ USC*γ*	-0.69	0.64	6	0.31	707.65	5
ASV_17717	p_ *Acidobacteria*; c_ *Acidobacteria*_Gp16; o_ Gp16	0	1	1	1	0	10
ASV_17791	p_ *Acidobacteria*; c_ *Acidobacteria*_Gp16; o_ Gp16	0	1	1	1	0	9
ASV_18813	p_ *Acidobacteria*; c_ *Acidobacteria*_Gp16; o_ Gp16	-1.70	1	1	0.24	0	1
ASV_18814	p_ *Acidobacteria*; c_ *Acidobacteria*_Gp16; o_ Gp16	0	0.78	3	0.21	1826	3
ASV_1895	p_ *Proteobacteria*; c_ *Alphaproteobacteria*; o_ *Rhizobiales*; f_ *Rhodobiaceae*	0	1	1	1	0	9
ASV_19050	p_ *Acidobacteria*; c_ *Acidobacteria*_Gp16; o_ Gp16	0	1	1	1	0	10
ASV_21687	p_ Others	0.96	0.69	6	0.26	2173.72	4
ASV_2254	p_ *Proteobacteria*; c_ *Gammaproteobacteria*; o_ *Chromatiales*; f_	-0.82	0.64	6	0.27	373.45	5

	unclassified_*Chromatiales*; g_ USC*γ*						
ASV_23444	p_ Others	-1.69	0.75	2	0.25	0	1
ASV_23473	p_ Others	-2.09	1	1	0.21	0	0
ASV_23899	p_ Others	-0.95	0.75	2	0.21	0	5
ASV_23927	p_ Others	0	0.75	2	0.18	0	3
ASV_2429	p_ *Proteobacteria*; c_ *Betaproteobacteria*	-0.95	0.75	2	0.21	0	5
ASV_2639	p_ *Proteobacteria*; c_ *Gammaproteobacteria*; o_ *Chromatiales*	-1.09	1	1	0.22	0	6
ASV_3345	p_ *Proteobacteria*; c_ *Betaproteobacteria*	-0.69	0.63	7	0.29	151.35	6
ASV_4122	p_ *Proteobacteria*; c_ *Gammaproteobacteria*; o_ *Chromatiales*; f_ unclassified_*Chromatiales*; g_ USC*γ*	-0.55	0.68	9	0.33	2755.48	5
ASV_5071	p_ *Proteobacteria*; c_ *Gammaproteobacteria*; o_ *Chromatiales*; f_ unclassified_*Chromatiales*; g_ USC*γ*	-0.24	0.64	6	0.26	45.66	4
ASV_5575	p_ *Proteobacteria*; c_ *Gammaproteobacteria*; o_ *Chromatiales*; f_ unclassified_*Chromatiales*; g_ USC*γ*	-0.69	0.71	7	0.30	776.42	5
ASV_5887	p_ *Proteobacteria*; c_ *Gammaproteobacteria*; o_ *Chromatiales*; f_ unclassified_*Chromatiales*; g_ USC*γ*	-0.95	0.75	2	0.22	0	5
ASV_5910	p_ *Proteobacteria*; c_ *Gammaproteobacteria*	-1.70	1	1	0.24	0	1
ASV_6140	p_ *Proteobacteria*; c_ *Alphaproteobacteria*; o_ *Rhizobiales*	0	0.75	2	0.18	0	3
ASV_6175	p_ *Proteobacteria*; c_ *Gammaproteobacteria*; o_ *Chromatiales*	-1.70	1	1	0.26	0	1

ASV_6409	p_ *Proteobacteria*; c_ *Gammaproteobacteria*; o_ *Chromatiales*	-0.69	0.64	6	0.29	2463.15	5
ASV_6656	p_ *Proteobacteria*; c_ *Gammaproteobacteria*; o_ *Chromatiales*; f_ unclassified_*Chromatiales*; g_ USC*γ*	-2.05	0.63	7	0.2	1442.05	5
ASV_673	p_ *Proteobacteria*; c_ *Gammaproteobacteria*	-1.70	1	1	0.23	0	1
ASV_7189	p_ *Proteobacteria*; c_ *Alphaproteobacteria*; o_ *Rhizobiales*	-0.69	0.68	9	0.29	2030.94	5
ASV_7670	p_ *Parcubacteria*	-1.70	1	1	0.24	0	1
ASV_7833	p_ *Nitrospirae*; c_ *Nitrospira*; o_ *Nitrospirales*; f_ *Nitrospiraceae*; g_ *Nitrospira*	-2.08	0.75	2	0.21	0	0
ASV_7909	p_ *Nitrospirae*; c_ *Nitrospira*; o_ *Nitrospirales*; f_ *Nitrospiraceae*; g_ *Nitrospira*	-0.82	0.69	4	0.26	12.64	6
ASV_8577	p_ *Gemmatimonadetes*; c_ *Gemmatimonadetes*; o_ *Gemmatimonadales*; f_ *Gemmatimonadaceae*; g_ *Gemmatimonas*	-0.95	0.75	2	0.22	188.55	6
ASV_8911	p_ *Firmicutes*; c_ *Bacilli*; o_ *Bacillales*; f_ *Bacillaceae*_1; g_*Bacillus*	-2.08	0.75	2	0.18	0	0
ASV_9022	p_ *Firmicutes*; c_ *Bacilli*; o_ *Bacillales*; f_ *Bacillaceae*_1; g_*Bacillus*	-1.43	0.75	2	0.23	0	4
ASV_9029	p_ *Firmicutes*; c_ *Clostridia*	-2.08	0.75	2	0.26	0.05	0
